# Pre-Clinical Assessment of Immune Responses to Adeno-Associated Virus (AAV) Vectors

**DOI:** 10.3389/fimmu.2014.00028

**Published:** 2014-02-07

**Authors:** Etiena Basner-Tschakarjan, Enoch Bijjiga, Ashley T. Martino

**Affiliations:** ^1^Department of Pediatrics, Children’s Hospital of Philadelphia, Philadelphia, PA, USA; ^2^Department of Pharmaceutical Sciences, St. John’s University, Queens, NY, USA

**Keywords:** immune responses, adeno-associated virus, pre-clinical models, AAV, inhibitory AAV

## Abstract

Transitioning to human trials from pre-clinical models resulted in the emergence of inhibitory AAV vector immune responses which has become a hurdle for sustained correction. Early animal studies did not predict the full range of host immunity to the AAV vector in human studies. While pre-existing antibody titers against AAV vectors has been a lingering concern, cytotoxic T-cell (CTL) responses against the input capsid can prevent long-term therapy in humans. These discoveries spawned more thorough profiling of immune response to rAAV in pre-clinical models, which have assessed both innate and adaptive immunity and explored methods for bypassing these responses. Many efforts toward measuring innate immunity have utilized Toll-like receptor deficient models and have focused on differential responses to viral capsid and genome. From adaptive studies, it is clear that humoral responses are relevant for initial vector transduction efficiency while cellular responses impact long-term outcomes of gene transfer. Measuring humoral responses to AAV vectors has utilized *in vitro* neutralizing antibody assays and transfer of seropositive serum to immunodeficient mice. Overcoming antibodies using CD20 inhibitors, plasmapheresis, altering route of delivery and using different capsids have been explored. CTL responses were measured using *in vitro* and *in vivo* models. In *in vitro* assays expansion of antigen-specific T-cells as well as cytotoxicity toward AAV transduced cells can be shown. Many groups have successfully mimicked antigen-specific T-cell proliferation, but actual transgene level reduction and parameters of cytotoxicity toward transduced target cells have only been shown in one model. The model utilized adoptive transfer of capsid-specific *in vitro* expanded T-cells isolated from immunized mice with LPS as an adjuvant. Finally, the development of immune tolerance to AAV vectors by enriching regulatory T-cells as well as modulating the response pharmacologically has also been explored.

## Introduction

Over the past 20 years, a full spectrum of immune responses to AAV has been assessed to include innate responses, humoral responses, and T-cell responses. In addition to the emphasis on pre-existing antibodies to AAV, cytotoxic T-cell (CTL) responses to AAV have emerged as a challenge for clinical applications. Roughly 70% of the human population has neutralizing antibodies (NAb) titers to AAV2 that can interfere with gene therapy (1–4). The use of different AAV capsids may overcome this challenge yet lower antibody titers to alternative capsids as well as cross-reactivity of AAV2 antibodies is concerning (3, 4).

Since mice and dogs do not have pre-existing immunity to AAV, correction in coagulation actor IX (FIX) deficient animal studies have been sustained without concern of immune responses (5–7). Unfortunately, the lack of immunity in pre-clinical studies did not fully reflect clinical results. In patient studies using AAV2 or AAV8 expressing normal or self-complimentary (sc) hFIX respectively, AAV1 expressing α1-antityrpsin (AAT), and Lipoprotein Lipase (LPL) correction, capsid-specific CTL responses have emerged, particularly at high doses (8–11). In some cases, existence of capsid-specific CTLs did not significantly impact correction as seen in AAT studies involving intramuscular (IM) injections (10). In hemophilia B (HB) liver studies, correction at 10% of normal was achieved in high dose cohorts. This level was only sustained 4–8 weeks and a spike in liver transaminases coincided with a drop in hFIX levels (8, 9), which was coordinated by capsid-specific CTLs. With the occurrence of CTL responses and presence of pre-existing antibodies to AAV capsid there has been a concerted effort to develop suitable pre-clinical approaches for complete assessment of immune responses and to develop intervention techniques.

## Characterization of Innate Immunity to AAV

Pattern recognition receptors (PRRs) on monocytes recognize AAV particles and trigger innate immunity (12–15). TLR2 and TLR9 have been identified as the PRRs for AAV. TLR2 triggers responses to the capsid while TLR9 detects the viral genome. Studies have been performed in myeloid differentiation primary response 88 (*myd88*−/−), *tlr9*−/− and *tlr2*−/− deficient mice to pinpoint TLR pathways associated with AAV recognition. Additionally, adaptive responses have been assessed in these mice. Moreover, studies were performed in interferon β receptor of KO mice (*ifnr*−/−) to establish a cytokine for developing adaptive responses (13, 14). Measuring cytokine secretion from differentiated monocytes isolated from these KO mice was used to segregate responses to AAV2. Absence of type I interferon (IFN) responses in AAV-activated plasmacytoid dendritic cells (pDCs) isolated from *tlr9*−/− mice as well as *myd88*−/− but not *trl2*−/− mice suggests that IFN responses are due to the vector genome, not the capsid (12–14).

To further assess the immune responses to AAV in KO mice, cellular and humoral responses were measured. Assessing NAbs, serum IgG2a or CTL responses to AAV capsid in mice deficient in innate immunity was used to determine a pathogen recognition link between innate and adaptive response. Both *tlr9*−/− and *myd88*−/− mice showed absence of adaptive responses to AAV (13, 14). AAV antibody titers were assessed by enzyme-linked immunosorbent assay (ELISA) while CTL responses were assessed using enzyme-linked immunosorbent spot (ELISpot) analysis of IFN-γ secretion from isolated splenocytes.

Efforts to determine if genome or capsid modifications alter innate responses have been pursued. Gene expression in liver of AAV2-infected mice showed an increase in inflammatory mediators and TLR9 transcripts, but not TLR2. Additionally, the responses remained the same between capsid variants but the sc genome resulted in a significant increase in transcription of inflammatory mediators. Determining cellular recruitment also indicated the sc-genome significantly increased inflammation. This was measured by staining liver sections for neutrophils, Mac-1+ macrophages and cd335+ natural killer cells. The use of *tlr9*−/− mice as well as TLR9 antagonists prevented innate immune responses, showed a reduction in adaptive immunity, and demonstrated a plausible connection between TLR9 and development of antibodies and CTLs (14).

Efforts to isolate immune responses to AAV input capsid have also been performed. TLR2 and TLR4 are highly associated with recognition of viral structural proteins (16). The production of empty capsids devoid of genomes and the use of HEK293 reporter cell lines, which secrete IL-8 due to activation of stably expressed TLR2 or TLR9, have aided in pinpointing PRRs associated with capsid. HEK293/TLR2 but not HEK293/TLR9 showed activation by empty vectors (15).

## Humoral Immunity

Epidemiology studies have predicted that ~70% of the population is seropositive for AAV2 (1–4). Further studies have estimated a lower degree of seroprevalence for other AAV capsids (4). Moreover, 20–40% of the population has NAb titers (1:80) against AAV2 capable of reducing transduction of Huh7 cultured cells by 50% using a Multiplicity of Infection of 10^4^. Additionally, the ability of AAV2 NAb to reduce transduction efficiency against alternative serotypes has developed concern for cross-reactivity.

## Characterizing NAb Inhibition of AAV

Dot Blot studies, NAb assays and cross administration of AAV vectors in animals have been used to determine cross-reactivity of AAV antibodies. The cross-reactivity of αAAV1 or αAAV5 antibodies was tested against a dot blot containing AAV1, AAV2, AAV4, AAV5, AAV6, AAV7, AAV8, AAV9 capsid. AAV1 antibodies showed strong adherence to only AAV6, while AAV5 antibodies had minimal adherence to AAV1 (17) and only at high concentrations of monoclonal antibodies.

In NAb *in vitro* assays, AAV vectors are pre-incubated with serum containing NAbs prior to infection of cultured cells and can be used for assessing cross-reactivity when alternative capsids are selected. Using this assay, it was determined that NAbs against AAV1 or AAV5 had limited impact on transduction from the other capsid. Additionally, engineered capsid modifications to AAV2 or a chimeric AAV capsid overcame antibody neutralization based on parental AAV antigens (4, 17–19).

Neutralizing antibody in mice also determined the potential of AAV antibodies to cross react with a different capsid using *in vivo* approaches. Mice were pre-immunized by IM injection of AAV2/GFP then challenged with AAV1, AAV3, AAV4, and AAV5 (20). Data showed that AAV2 antibodies only neutralized AAV3. Another model used an intravenous (IV) infusion in mice of AAV2 immunoglobulin from a pool of individual human donors prior to liver delivery of AAV2, AAV6 or AAV8 expressing hFIX. AAV2 administration was completely blocked while AAV6 and AAV8 were partially blocked (18). Given that the immunoglobulin pool was not assessed for AAV6 or AAV8 antibodies the results seen may not have been due to cross-reactivity.

## Overcoming Humoral Responses to AAV Vectors

A comparative approach to test the ideal route of administration to overcome pre-existing NAbs in mice involved an initial IV infusion of AAV2 immunoglobulin from a collection of AAV2 seropostive serum prior to administrating AAV2 via different routes. Delivery of AAV2 to liver by portal vein or direct injection into the liver parenchyma showed successful transduction in the face of pre-existing AAV2 antibodies prophylaxis, while IV injection did not (21). It should be noted that even direct administration of vector to the liver resulted in lower expression levels in the presence of NAb, indicating that even this approach is not able to completely bypass Nab inhibition.

Manipulating the capsid is another approach to overcome NAbs. Generation of alternative capsid libraries by rational mutations in antigenic regions (22), error prone PCR (23), and DNA shuffling (24) have demonstrated the potential. PEGylation is a chemical modification which involves pre-coating the AAV capsid with polyethylene glycol (PEG) prior to administration and has shown promise at limiting but not preventing neutralization (25, 26). Another promising strategy involves artificially encapsulating the AAV vector in biomaterial prior to administration which has the potential to shield the AAV capsid (27). This may be useful provided the biomaterial cloak can be degraded after delivery. While these approaches are encouraging they may limit transduction efficiency.

Temporary immunosuppresion (IS) is emerging as a preferred approach for overcoming humoral responses and has been tested in pre-clinical studies. The inhibition of helper T-cells using CD4 antibodies or cyclosporine A prevents NAb formation and facilitates vector readministration (28). Additionally, transient B-cell depletion using Rituximab (CD20 antibody) can reduce pre-existing antibodies (29). Plasmapheresis can eliminate pre-existing humoral responses but requires multiple cycles of blood exchange to reduce NAbs to negligible levels (30). Similarly, flushing the macaque liver with saline prior to delivery of vector limited NAb inhibition (31). In an ongoing clinical trial, empty capsids were added to the vector with the rational that they will bind away Nabs and thus increase transgene expression (32). However this approach involves increasing vector load, which may not be favorable for trying to prevent the development of CTL responses.

## Characterizing Inhibitory CTL Responses to AAV in Pre-Clinical Studies

The first CTL response with parenchymal damage, loss of transgene expression, and expansion of capsid-specific CTLs were observed during a liver directed clinical trial for HB. This was unexpected since it was not observed in pre-clinical studies including non-human primates. Early attempts to replicate this immune response in animals were unsuccessful (33, 34). Even dog models have shown consistent transgene expression without an immune response (5). Therefore, efforts were made to determine variations in immunity between humans and pre-clinical animals. The absence of CTL responses in non-human primates, which are natural hosts of AAV8, ruled out the expansion of memory T-cells as the contributing factor (35, 36). Varki and colleagues reported the loss of siglec receptor expression in T lymphocytes during human evolution (37). Siglec receptors have inhibitory function on the immune system. Exploring the loss of these inhibitory receptors as a possible reason revealed enhanced T-cell function in a mouse KO model which mimics human T-cells, suggesting that human T-cells respond with more activation and proliferation (38). However, the response to AAV also could not be induced in these KO mouse.

In trying to develop CTL responses in pre-clinical animal models, one approach delivered a rAAV expressing IL-12 to the liver (39). Despite the induction of liver inflammation and generation of capsid-specific CTLs, transduced hepatocytes were not eliminated. Another model used an immunogenic OVA/SIINFEKL mouse system. The OT-1 mouse line has an increased frequency of OVA SIINFEKL epitope-specific CTLs. The model requires immunization of OT-1 mice with Ad-AAV. Splenocytes were harvested, labeled with carboxyfluorescein succinimidyl ester (cfse), and adoptively transferred into mice injected with an AAV vector with the SIINFEKL epitope incorporated into the capsid (40). Prolonged proliferation of OT-1 transferred cells (at least 10 weeks after transfer) was observed compared to transfer of wt splenocytes from Ad-AAV immunized mice, however no parenchymal cytotoxicity was reported. The Samulski lab also used the SIINFEKL mouse system. They discovered reduced transgene expression after adoptive transfer of OT-1 splenocytes however only in AAV-treated mice pre-immunized with SIINFEKL-pulsed DCs. Unfortunately, this approach is not ideal given the high immunogenicity, which might not fully reflect actual immunity (41).

## *In vitro* Assays for Characterizing CTLs

*In vitro* assays have had more success at characterizing AAV capsid CTL development and involve monitoring CTL activation and cytotoxicity in response to peptide presentation of AAV capsids in target cells. It is possible to detect AAV capsid-specific CTLs by staining with a multimeric antibody. However staining with these multimers only detects single T-cell clones and is HLA restrictive. As a result, T-cells only from individuals who express a specific HLA type can be visualized. An engineered cell line, which expresses luciferase upon engagement of a specific T-cell receptor was used to monitor T-cells activation (42). Again, this assay is restricted to one HLA type (HLAB0702) and AAV2 recognition. A more flexible assay measures cytotoxicity toward AAV-transduced hepatocytes. In this assay, different serotypes can be tested as long as they transduce the target cells (43, 44). Briefly, CTLs are generated by several rounds of stimulation and proliferation of target cell HLA matched peripheral blood mononuclear cells (PBMCs) or spenocytes with the AAV serotype of interest or dominant epitope and then co-cultured with target cells that are transduced with that AAV and cytotoxicity is measured. In general, a major limitation of *in vitro* assays is the fact that transduction efficiency of alternate AAV serotypes differs significantly and hinders comparative studies between alternate capsid variants and serotypes. Still these assays are helpful in monitoring CTLs responses in mice and in PBMCs obtained during clinical trials.

## Vector Capsid Variations for Avoiding CTL Responses

There is a growing field of vector alterations to increase efficiency and avoid immunity. The rational for creating capsid mutants differs among groups, however most target tyrosine and serine residues on the AAV capsid to enhance transduction efficiency. The Srivastava lab (45) has reported a triple tyrosine capsid mutant with increased transduction efficiency *in vitro* and *in vivo*. Other groups also report increased transduction efficiency by altering tyrosine residues on the AAV capsid (46). Initial data suggests that increased transgene expression is likely due to reduced proteasomal degradation which will shuttle more vector to the nucleus, but the intracellular trafficking of vector has not been fully characterized.

From an immunological perspective, avoiding proteasomal degradation may also lead to reduced CTL recognition. Tyrosine mutants were tested in an immunological mouse model and the group reported increased transgene expression and showed reduced T-cell immunogenicity and toxicity within the liver (44). To mimic the cellular immune reaction in humans, the group adoptively transferred *in vitro* expanded CTLs from AAV immunized mice. In this setting, they were able to demonstrate cytotoxicity in the liver by reduction of transgene levels and increased liver enzymes, both of which were reversed when using the capsid mutant.

## Pharmacological Intervention

General IS (taken from treatment of autoimmune disorders or transplantations) (9) and more specific interventions for AAV have been proposed and tested in pre-clinical models, such as proteasomal inhibitors (PI) (e.g., bortezomib, MG 132, carfilzomib). Many groups focus on increasing AAV transduction levels in various tissues using PI (47–50), and other compounds like arsenic trioxide (51), rather than their influence on the immune response. At the same time, another group observed reduced CTL responses as well (42). The mechanism is not completely understood yet it is hypothesized that AAV proteasomal capsid degradation is disrupted which leads to diminished capsid–peptide presentation by MHC class I molecules. This intervention has only been tested in pre-clinical systems for mechanistic value, given that the later fate of undigested capsids is unknown and could present the danger of prolonged or delayed capsid–peptide presentation.

## Developing Tolerance

Multiple groups have demonstrated induced tolerance to the transgene for hepatic delivery of AAV vectors with the involvement of regulatory T-cells (Tregs) (52–55). Attempts to induce tolerance to the AAV capsid through the enrichment of Tregs have been published and show promising results. Initially, De Groot and colleagues were able to show reduced immune responses to common immunogens *in vitro* and *in vivo* using MHC class II epitopes (Tregitopes) that favor Treg development (56). These observations were extended to AAV capsid tolerance in a subsequent study (57). They show AAV capsid CTL suppression using these Tregitopes.

## Conclusion

Due to the combined efforts of multiple groups working on the various aspects of immune responses to gene delivery with a rAAV vector, many parameters have been deciphered. For humoral immune response models, various models have been established to characterize pre-existing humoral immunity and cross-reactivity and assess the impact of potential interventions *in vitro* and *in vivo*. Using pre-existing KO mice, the influence of the innate immune system is also being rapidly investigated. In contrast, finding models to mimic and investigate the cellular immune response toward AAV capsids has caused more difficulty, only robust *in vitro* models have been well established but recently some features of the response could be recapitulated in animal models. It remains to be seen if these approaches will be able to provide a sufficient platform for further deciphering of cellular immune response and testing interventions. While characterizing immune responses in animal models have been informative, it is evident that greater emphasis should be placed on developing CTL models in immunocompetent animals that are more reflective of the clinical scenario. Until reliable models are established, investigators can use the valuable published data gathered in human clinical trials about cellular immune responses and their management (8, 9).

## Conflict of Interest Statement

The authors declare that the research was conducted in the absence of any commercial or financial relationships that could be construed as a potential conflict of interest.
